# Damage accrual and predictors of mortality in ANCA-associated vasculitis: a retrospective observational study

**DOI:** 10.1007/s00296-025-05883-7

**Published:** 2025-05-07

**Authors:** Murat Bektaş, Burak Ince, Sibel Zaralı, Übeyde Ayşe Gülseren, Ece Ük, Besim Fazıl Ağargün, Damla Yenersu Güzey, Yasemin Yalçınkaya, Bahar Artım-Esen, Ahmet Gul, Murat İnanç

**Affiliations:** 1 Department of Internal Medicine, Division of Rheumatology Istanbul Faculty of Medicine, Istanbul, Türkiye; 2 Department of Internal Medicine, Istanbul Faculty of Medicine, Istanbul, Türkiye; 3 Istanbul Faculty of Medicine, Istanbul, Türkiye; 4 Department of Internal Medicine, Division of Rheumatology, Istanbul Aydın University, Istanbul, Türkiye

**Keywords:** Anti-Neutrophil Cytoplasmic Antibody (ANCA), Associated Vasculitis (AAV), Granulomatous with polyangiitis (GPA), Microscopic polyangiitis (MPA), Vasculitis, Cardiovascular diseases, Malignancy

## Abstract

In this study, we aimed to evaluate the factors affecting the development of damage and mortality in patients with AAV treated at our tertiary referral center. This retrospective study included data on patients with AAV who fulfilled the Chapel Hill Consensus Conference (CHCC) criteria. Patients were divided into c-ANCA/PR3( +) and p-ANCA/MPO ( +) groups based on ANCA immunofluorescence and/or ELISA results, and relapse, damage, and mortality data were compared across the groups. Data from 254 patients (n = 136, 53.5% female) were included in the analysis. Clinical diagnosis was GPA in 186 (73.2%) and MPA in 68 (26.8%) patients. During the follow-up, 217 of 242 (89.7%) patients developed damage, and the median VDI score of the cohort was 2 (IQR: 2). VDI scores were higher in the first period (1997–2011) than in the second period (2011–2021) in the entire cohort (p = 0.012) and in patients with GPA compared with MPA (p = 0.034). Five-year and overall survival rates were 88.1% and 80.3% in the entire cohort; 87.8% and 81% in c-ANCA/PR3 ( +); 86.2% and 76% in p-ANCA/MPO ( +) (Log-Rank: p = 0.35); 91% and 84.5% in GPA; 81% and 67.2% in MPA patients (Log-Rank: p < 0.001). Development of malignancy, severe infection, and active/persistent disease after the induction phase were associated with higher mortality in patients with AAV. In our AAV cohort, permanent organ damage was detected in the majority of the patients. Although the median VDI score decreased over time, mortality did not change.

**Supplementary Information:**

The online version contains supplementary material available at 10.1007/s00296-025-05883-7.

## Introduction

Antineutrophil cytoplasmic antibody (ANCA)-associated vasculitis is characterized by inflammation of small-sized blood vessels and encompasses several clinicopathological entities, including granulomatosis with polyangiitis (GPA), microscopic polyangiitis (MPA), and eosinophilic granulomatosis with polyangiitis (EGPA). Although these entities differ in pathogenesis, genetics, and serotypes, severe forms of ANCA-associated vasculitis (AAV) share several clinical features and currently have similar treatments. The introduction of immunosuppression with cyclophosphamide (CYC) and corticosteroids (CS) significantly improved prognosis, reducing mortality from 80% at one year in untreated patients to 90% of patients achieving remission [1]. While rituximab (RTX) or CYC was the standard induction treatment regimen of patients who had major organ involvement in AAV [2], in the maintenance phase, RTX, azathioprine (AZA), mycophenolate mofetil (MMF), and methotrexate (MTX) are the treatment options.

The leading causes of mortality in AAV are active vasculitis and infection during the induction phase, cardiovascular disease (CVD), and malignancy during the maintenance phase [1, 3]. Several prognostic scores are available for predicting mortality in AAV, and the most widely used one is the five-factor score (FFS), which identifies higher age (> 65 years), gastrointestinal, cardiac involvement, and renal involvement as poor prognostic factors [4]. Although the outcomes of patients with AAV have improved significantly over the past years, a significant proportion of patients are still facing the risk of relapse, organ damage, and mortality [5]. Although the relapse rates were found to be higher in patients with GPA, those with MPA had higher mortality and end-stage renal disease (ESRD) [6].

In this study, we aimed to evaluate the demographic and clinical characteristics of patients with AAV in our tertiary referral center according to ANCA status, relapse, damage, and mortality data.

## Material and methods

### Patients and data

We retrospectively evaluated the patients who were followed up in our vasculitis clinic in Istanbul, Türkiye, between 1997 and 2021. Patients who fulfilled the Chapel Hill Consensus Conference (CHCC) criteria were included in the study [7]. Patients with missing data or patients who did not meet the CHCC criteria were excluded. Patients with EGPA were excluded from the study due to different clinical trajectories and treatment modalities from GPA and MPA. Clinical data were collected with standardized forms from patients’ charts. ANCA results obtained with immunofluorescence and/or ELISA were recorded. Patients were divided into two serological groups based on ANCA positivity: c-ANCA/PR3 ( +) and p-ANCA/MPO ( +).

Institutional Review Board approval was obtained from the Istanbul Faculty of Medicine Ethics Committee for this study (date and number: 06/07/2020-75926).

### Definition of disease activity, damage, and outcome

In this study, disease activity was evaluated by the Birmingham Vasculitis Activity Score version 3 (BVAS v3) and calculated retrospectively [8]. Remission was defined as the absence of clinical signs or symptoms attributed to AAV with or without immunosuppressive therapy after the induction phase (6th month) [9]. Relapse was defined as the recurrence of active disease following a period of remission with an increased need for immunosuppressive treatment with compatible clinical and laboratory findings [9]. Severe infection was defined as the development of opportunistic infection and/or the need for hospitalization, intravenous antibiotics, sepsis, or intensive care unit requirement. Permanent organ damage was recorded according to the vasculitis damage index (VDI) [10]. Cardiovascular events (CVE) were defined as a history of unstable angina pectoris, myocardial infarction (MI), peripheral artery disease, and need for revascularization such as angioplasty or surgery. Cerebrovascular accident (CVA) was not included in the definition of CVE and was evaluated separately. Venous thromboembolism (VTE) was defined as deep venous thrombosis (DVT) and/or pulmonary thromboembolism. The estimated glomerular filtration rate (eGFR) was calculated by the CKD-EPI formula [11]. The development of ESRD was defined as the need for renal replacement therapy such as dialysis or renal transplantation. Low GFR status was defined as eGFR < 50 ml/min as in the VDI score [10]. VDI was evaluated in two different periods of diagnosis, before and after 2011 (First period) and (Second period), because of the approval of RTX in 2011 in AAV. Mortality information was checked and confirmed by the National Death Notification System (OBS).

### Statistical analysis

In our study, the 21.0 version (IBM, Armonk, NY, USA) of the SPSS (Statistical Package for the Social Sciences) program was used for the statistical analysis of data. In descriptive statistics, discrete and continuous numerical variables were expressed as mean, ± standard deviation, or median and interquartile range (IQR). Categorical variables were expressed as number of cases (%). Cross-table statistics were used to compare categorical variables (Chi-Square, Fisher’s exact test). Normally distributed parametric data were compared with Student’s t-test, and non-parametric data that did not meet normal distribution were compared with Mann Whitney U and Kruskal Wallis tests. Correlation analysis was performed using the Pearson or Spearman method, depending on the normality of the distribution. Kaplan–Meier and log-rank methods were used for survival analysis. Multivariate analysis was performed by using logistic regression. P < 0.05 value was considered statistically significant.

## Results

### Baseline clinical and laboratory features of patients

We evaluated the records of 320 patients with AAV in our study. Of those, data from 28 patients with EGPA, 25 patients who had missing data, and 13 patients who did not fulfill the CHCC criteria were excluded, and 254 patients (n = 136, 53.5% female) were included in the analysis. The mean age of diagnosis was 55.6 ± 14.1 (range: 17–88) years, and the median disease duration was 67.5 (IQR: 77) months. Clinical diagnosis was GPA in 186 (73.2%) and MPA in 68 (26.8%) patients. ANCA results were available in 244 patients; 131 (53.7%) patients were c-ANCA/PR3 ( +), 88 (36.1%) patients were p-ANCA/MPO ( +), and 25 (10.2%) patients were ANCA negative. The most frequent organ involvements were the kidney (75.8%) and lower respiratory tract (LRT) (74.4%). The baseline clinical and laboratory characteristics of the patients are provided in Table 1. The mean baseline BVAS score was 17 ± 6.9 at admission (range, 3–40) and was similar between the two serological groups (Table 1).

**Table 1:** Baseline clinical and laboratory features of the patients with ANCA-associated vasculitis

Variables	Total (n = 254)	c-ANCA/PR3^+^ (n = 131)	p-ANCA/MPO^+^ (n = 88)	p-value (OR)
Age, years, mean ± SD, range	55.6 ± 14.1 (17–88)	52.5 ± 13.2 (23–81)	60 ± 14.9 (17–88)	** < *0.001***
Gender, female, n (%)	136 (53.5)	57 (43.5)	56 (63.6)	***0.003 (8.5)***
Treatment duration (months), median (IQR)	67.5 (77)	60 (79)	60 (84)	0.8*
Diagnosis, n (%)				
GPA	186 (73.2)	131 (70.4)	25/186 (13.4)	
MPA	68 (26.8)	0	63/68 (92.6)	
Organ involvement, n (%)				
Upper respiratory tract	140/246 (56.9)	98/129 (76)	21/85 (24.7)	** < *0.001 (54.5)***
Ear nose throat	64/244 (26.2)	48/128 (37.5)	10/85 (11.8)	** < *0.001 (17.1)***
Eye	40/246 (16.3)	32/129 (24.8)	4/86 (4.7)	** < *0.001 (15)***^***Ɨ***^
Lower respiratory tract	186/250 (74.4)	105/129 (81.4)	60/88 (68.2)	***0.025 (5)***
Alveolar hemorrhage	33/247 (13.4)	15/129 (11.6)	16/87 (18.4)	0.16
Interstitial lung disease	44/192 (22.9)	18/96 (18.8)	24/75 (32)	***0.046 (4)***
Heart	16/248 (6.5)	9/131 (6.9)	7/87 (8)	0.7^***Ɨ***^
Kidney involvement	191/252 (75.8)	100/131 (76.3)	74/88 (84.1)	0.16
Central nervous system	16/248 (6.5)	8/131 (6.1)	6/87 (6.9)	0.8^***Ɨ***^
Peripheral nervous system	51/247 (20.6)	24/130 (18.5)	20/87 (23)	0.4
Mononeuritis multiplex	24/246 (9.8)	12/130 (9.2)	9/87 (10.3)	0.8^***Ɨ***^
Gastrointestinal system	13/248 (5.2)	9/131 (6.9)	2/87 (2.3)	0.13^***Ɨ***^
Skin	55/246 (22.4)	35/129 (27.1)	11/87 (12.6)	***0.01 (6.5)***
Arthritis	79/214 (36.9)	62/129 (48.1)	17/85 (20)	***<0.001 (17.3)***
BVAS score, mean ± SD, range	17 ± 6.9 (3–40)	17.5 ± 7.2 (5–40)	17.5 ± 5.7 (7–30)	1
Smoking history (ever), n (%)	59/219 (26.9)	38/115 (33)	17/78 (21.8)	0.09
Nasal staph aureus carriage, n (%)	36/206 (17.5)	24/109 (22)	6/76 (7.9)	***0.01 (6.6)***^***Ɨ***^
Baseline leucocyte levels, median (IQR)	10,600 (6748)	11,100 (5320)	9400 (7185)	0.2*
Baseline hemoglobin levels, median (IQR)	10.2 (3.1)	10.3 (4)	9.95 (2.8)	0.2*
ESR (mm/hour), median (IQR)	86 (63)	91.5 (53)	86 (61)	0.2
C-reactive protein (mg/L), median (IQR)	56.5 (95)	66 (98)	48 (95)	0.042*
Baseline creatinine levels, median (IQR)	1.5 (2.5)	1.15 (2.1)	2.2 (3.1)	0.005*
RF positivity, n (%)	49/101 (48.5)	30/58 (51.7)	15/29 (51.7)	1
Cumulative steroid (MP) dose (g/12 month), median (IQR)	7.5 (8)	8 (7.5)	5 (30)	0.3*
Cumulative CYC dose (g), median (IQR)	4.5 (7.5)	5.5 (9)	4 (5.7)	0.043*
Relapse, n (%)	81/218 (35.1)	51/120 (42.5)	24/83 (28.9)	***0.049 (3.9)***
Severe infection, n (%)	83/218 (38.1)	49/118 (41.5)	30/78 (38.5)	0.7
VDI score, median (IQR)	2 (2)	2 (3)	2 (2)	0.9*
Mortality, n (%)	50 (19.7)	24 (18.3)	21 (23.9)	0.3

*SD* Standard deviation, *IQR* Interquartile range, *GPA* Granulomatous with polyangiitis, *MPA* Microscopic polyangiitis, *BVAS* Birmingham vasculitis activity score, *ESR* Erythrocyte sedimentation rate, *RF* Rheumatoid factor, *MP* Methylprednisolone, *CYC* Cyclophosphamide, *VDI* Vasculitis damage index, *OR* Odds ratio

*Mann Whitney U test

^Ɨ^Fisher’s exact test

Bold and italic values are statistically significant (p < 0.05)

All patients received CS treatment during the induction phase. The median cumulative CS (methylprednisolone) dosage at 12 months was 7.5 g (IQR: 8) and was comparable across patients in the two serological subgroups (p = 0.3). CYC was used in 184 (79%), MTX in 23 (10%), RTX in 37 (16%), and MMF in 9 (4%) in the induction phase. The median cumulative CYC dosage was 4.5 g (IQR:7.5), which was higher in c-ANCA/PR3 ( +) patients compared to p-ANCA/MPO ( +) (p = 0.043). AZA was used in 110 (47.2%), RTX in 52 (22.3%), the combination of RTX and AZA in 18 (7.7%), MTX in 36 (15.5%), and MMF in 17 (7.3%) patients in the maintenance phase.

Remission status was evaluated in 174 patients, and 107 (64.1%) were found to be in remission after the induction treatment. Twenty-seven patients (16.2%) had active disease, 14 patients (8.4%) had persistent disease, and 19 patients (11.4%) were in partial remission at the 6th month. Relapses were observed in 81 of 218 patients (35.1%) and were higher in the c-ANCA/PR3 ( +) group compared to p-ANCA/MPO ( +) (42.5% vs 29%; p = 0.049, Odds ratio [OR]:3.9) and tended to be higher in patients with GPA compared to MPA (38.7% vs 25.8%; p = 0.07).

### Damage

#### VDI scores

During the follow-up, 217 of 242 (89.7%) patients developed damage, and the median VDI score of the cohort was 2 (IQR: 2). VDI scores were similar between serological subgroups (p = 0.9), also between GPA and MPA (p = 0.4). VDI score was correlated with baseline BVAS score (r = 0.247; p = 0.004), creatinine level at baseline (r = 0.218; p = 0.004), and higher age (r = 0.2; p = 0.002). No correlation was observed between the VDI score and the cumulative dose of CS or CYC. VDI scores were higher in the first period than in the second period in the entire cohort (p = 0.012) and in patients with GPA compared with MPA (p = 0.034). The cumulative CYC dose was also higher in the first period compared to the second period, as expected (p = 0.006). The relapse rate was also higher in the first period than in the second period (p = 0.02; OR:5.3) (Table 2). VDI score was higher in patients who died or relapsed (p = 0.001 and p < 0.001, respectively) (Table 3).

**Table 2:** Univariate analysis of clinical and laboratory features of the patients with ANCA-associated vasculitis according to the first and second periods

Variables	First period (n = 60)	Second period (n = 194)	p-value (odds ratio)
Age, years, mean ± SD	57.8 ± 13.6	55 ± 14.3	0.2
Gender, female, n (%)	28 (46.7)	108 (55.7)	0.2
Diagnosis, n (%)			
GPA	48 (80)	138 (71)	0.18
MPA	12 (20)	56 (29)	
Autoantibody status, n (%)			
c-ANCA/PR3^+^	29/50 (58)	102/169 (60.4)	0.8
p-ANCA/MPO^+^	21/50 (42)	67/169 (39.6)	
BVAS score at admission, mean ± SD	18.8 ± 7.3	16.5 ± 6.7	0.1
BVAS score at 6th month, mean ± SD	5.4 ± 3.8	4.2 ± 3	0.07
Presence of any VDI item, n (%)	55/58 (94.8)	162/184 (88)	0.14
VDI score, median (IQR)			
Total	3 (3)	2 (3)	0.012*
GPA	3 (3)	2 (3)	0.034*
MPA	4 (4)	2 (2)	0.064
Cumulative steroid (MP) dose, median (IQR)	7.7 (7.5)	7.5 (9.9)	0.5
CYC treatment at induction phase n (%)	48/55 (87)	136/180 (76)	0.065
Cumulative CYC dose, median (IQR)	12 (32)	5 (5.8)	0.006*
VDI items, n (%)			
eGFR < 50 at the last visit	21/58 (36.2)	62/180 (34.4)	0.8
ESRD	7/58 (12)	26/179 (14.5)	0.6
Cardiovascular events	11/58 (19)	27/179 (15)	0.5
Cerebrovascular accident	5/58 (8.6)	10/179 (5.6)	0.4
Avascular necrosis	13/57 (22.8)	22/177 (12.4)	0.056
Venous thrombosis	2/58 (3.4)	19/181 (10.5)	0.1
Malignancy	6/58 (10.3)	14/182 (7.7)	0.5
Remission at 6th month, n (%)	24/37 (64.9)	83/130 (63.8)	0.9
Relapse, n (%)	26/54 (48)	55/177 (31)	***0.02 (5.3)***
Severe infection, n (%)	17/47 (36.2)	66/171 (38.6)	0.8
Mortality, n (%)	7 (11.7)	43 (22.2)	0.074

*SD* Standard deviation, *IQR* Interquartile range, *GPA* Granulomatous with polyangiitis, *MPA* Microscopic polyangiitis, *BVAS* Birmingham vasculitis activity score, *MP* Methylprednisolone, *CYC* Cyclophosphamide, *RTX* Rituximab, *VDI*: Vasculitis damage index, *eGFR* Estimated glomerular filtration rate, *ESRD* End-stage renal disease, *OR* Odds ratio

*Mann Whitney U test

Bold and italic values are statistically significant (p < 0.05)

**Table 3:** Univariate and multivariate analysis of factors associated with mortality in patients with ANCA-associated vasculitis

	Univariate analysis			Multivariate analysis	
Variables	Exitus (n = 50)	Alive (n = 204)	p-value	CI % 95 (OR)	p-value
Age (years), mean ± SD	61.9 ± 14.3	54.07 ± 13.7	** < *0.001***		NS
Gender, female, n (%)	28 (56)	108 (52.9)	0.7		
Diagnosis, n (%)					
GPA (n = 186)	28 (15.1)	158 (84.9)	***0.002 (9.4)***		NS
MPA (n = 68)	22 (32.4)	46 (67.6)			
ANCA status, n (%)					
c-ANCA/PR3 (n = 131)	24 (18.3)	107 (81.7)	0.3		
p-ANCA/MPO (n = 88)	21 (23.9)	67 (76.1)			
Lung involvement, n (%)	38/186 (20.4)	11/64 (17.2)	0.6		
Kidney involvement, n (%)	42/191 (22)	7/61 (11.5)	0.09*		NS
eGFR < 50 ml/min, n (%)	21/83 (25.3)	21/155 (13.5)	***0.023 (5.1)***		NS
ESRD, n (%)	7/33 (21.2)	35/204 (17.2)	0.6		
Cardiovascular events, n (%)	11/38 (29)	31/199 (15.6)	***0.048 (3.9)***		NS
Cerebrovascular accident, n (%)	6/15 (40)	36/222 (16.2)	0.03 (5.5)*		NS
Avascular necrosis, n (%)	8/35 (23)	33/199 (16.6)	0.4		
Venous thrombosis, (%)	4/21 (19)	39/218 (18)	0.9		
Malignancy, n (%)	7/20 (35)	35/220 (16)	0.04 (4.6)*	***1.83–130 (15.5)***	***0.012***
BVAS score, mean ± SD, range	17.7 ± 4.2	16.8 ± 7.3	0.5		
Smoking history (ever), n (%)	15/59 (25.4)	24/160 (15)	0.074		NS
Baseline leucocyte levels, median (IQR)	10,900 (9050)	10,500 (6400)	0.8		
Baseline hemoglobin levels, median (IQR)	9.2 (1.8)	10.5 (3.8)	***0.015***		NS
ESR (mm/hour), median (IQR	98 (52)	77 (65)	0.15		
C-reactive protein (mg/L), median (IQR)	79 (108)	54 (93)	0.1		
Baseline creatinine levels, median (IQR)	2.3 (2.9)	1.4 (2.25)	0.24		
Relapse, n (%)	20/81 (24.7)	22/150 (14.7)	0.06		NS
Severe infection, n (%)	26/83 (31.3)	15/135 (11.1)	** < *0.001 (13.8)***	***1.04–29.2 (5.5)***	***0.045***
VDI score, median (IQR)	4 (3)	2 (3)	***0.001Ɨ***		NS
Remission at 6. Month, n (%)	9/107 (8.4)	18/60 (30)	0.001 (13.2)*	***1.7–21.3 (6)***	***0.005***

*SD* Standard deviation, *IQR* Interquartile range, *CI* Confidence interval, *GPA* Granulomatous with polyangiitis, *MPA* Microscopic polyangiitis, *ANCA* Anti-neutrophil cytoplasmic antibody, *eGFR* Estimated glomerular filtration rate, *ESRD* End-stage renal disease, *BVAS* Birmingham vasculitis activity score, *ESR* Erythrocyte sedimentation rate, *VDI* Vasculitis damage index, *OR* Odds ratio, *NS* Not significant

*Mann Whitney U test

^Ɨ^Fisher’s exact test

Bold and italic values are statistically significant (p < 0.05)

#### Individual VDI items

The frequency of patients with lower eGFR levels (eGFR < 50 ml/min) at the last visit and ESRD were 34.9% and 13.9%, respectively. While osteoporosis was higher (22.9% vs 12.3%, p = 0.045; OR:4) and asthma/chronic dyspnea (14.1% vs 6.6%, p = 0.07) tended to be higher in patients with p-ANCA/MPO ( +) compared to c-ANCA/PR3 ( +); cataract (6% vs 21.5%, p = 0.003; OR:9.1), hearing loss (2.4% vs 16.7%, p = 0.001; OR:10.5) and nasal septum perforation (2.4% vs 16.2%, p = 0.001; OR:10.2) were lower in p-ANCA/MPO ( +) group (Supplementary Table 1).

Overall, the development of CVE was 16% and tended to be higher in patients with c-ANCA/PR3 ( +) compared to those with p-ANCA/MPO ( +) (20.5% vs 10.6%; p = 0.06). VTE developed in 21 patients (8.8%), and avascular necrosis (AVN) was detected in 35 patients (15%), which were not different across the two serological groups. There was no association between AVN, cumulative dose of CS or CYC, and baseline BVAS score.

#### Malignancies

Twenty-two malignancies were observed in 20 patients (20/240, 8.3%; 11 c-ANCA/PR3 ( +), 3 in p-ANCA/MPO ( +), and 2 in ANCA negative patients), which were 3 carcinomas of the lung, 7 genitourinary tract, 5 hematological system, 3 thyroid papillary, 2 breast, 1 sarcoma and 1 oral cavity. There was no association between malignancy and cumulative dose of CYC or history of smoking. The frequency of malignancy did not differ between patients with GPA and MPA (8.6% vs 7.7%; p = 0.8). The development of CVE was also higher in patients who had malignancy (p = 0.003; OR: 11.7). The development of malignancy was frequent in patients who had higher BVAS scores at baseline (p = 0.049), and higher VDI scores (p = 0.02) in the univariate analysis. A significant association was observed between malignancy and CVE (95% confidence interval [CI]:2.2–83; OR:13.4, p = 0.005) in the multivariate analysis (Supplementary Table 2).

#### Mortality

In our cohort, fifty patients died (19.7%), including eight (16%) who were in the induction phase. Causes of mortality were available for 25 patients, and most of them were due to severe infection (n = 11; 44%). Five patients (20%) died due to active vasculitis, 5 patients (25%) from cancer, 2 patients (8%) from bowel perforation, one patient (4%) from bone marrow failure, one patient (4%) from heart failure, and one patient (4%) from a CVA.

Significantly higher mortality rate was observed in patients with older age (p < 0.001), MPA (p = 0.007; OR:7.3), history of severe infection (p < 0.001; OR:13.8), higher VDI score (p = 0.001), active/persistent disease at 6th month (p = 0.001; OR:13.2), in patients who developed CVE (p = 0.048; OR:3.9), CVA (p = 0.03; OR:5.5) malignancy (p = 0.04; OR:4.6), lower eGFR levels (eGFR < 50 ml/min) at last visit (p = 0.023; OR:5.1), and lower hemoglobin levels at baseline (p = 0.015) in the univariate analysis. Development of malignancy (95% CI:1.83–130; OR:15.5), severe infections (95% CI:1.04–29.2; OR:5.5), and active/persistent disease after the induction phase (95% CI:1.7–21.3; OR:6) were associated with higher mortality in the multivariate analysis (Table 3).

**Fig. 1:**
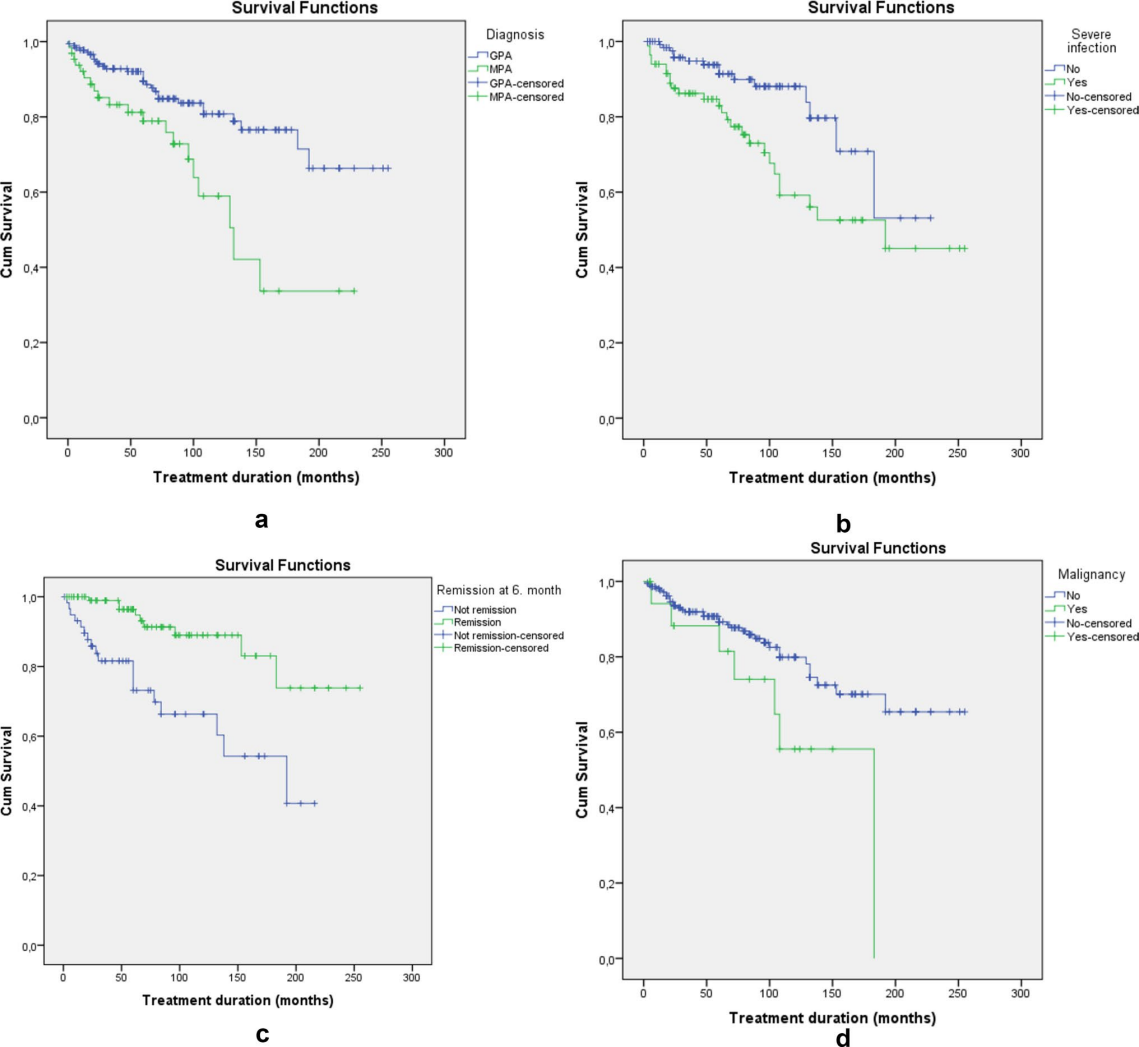
**A** Survival analysis of mortality rate according to clinical diagnosis in patients with ANCA-associated vasculitis. Log-Rank: p < 0.001. **B** Survival analysis of mortality rate according to presence of severe infection in patients with ANCA-associated vasculitis. Log-Rank: p = 0.004. **C** Survival analysis of mortality rate according to remission status after six months in patients with ANCA-associated vasculitis. Log-Rank: p < 0.001. **D** Survival analysis of mortality rate according to presence of malignancy in patients with ANCA-associated vasculitis. Log-Rank: p = 0.035

In our study, 5-year and overall survival rates were 88.1% and 80.3% in the entire cohort; 87.8% and 81% in c-ANCA/PR3 ( +); 86.2% and 76% in p-ANCA/MPO ( +) (Log-Rank: p = 0.35); 91% and 84.5% in GPA; 81% and 67.2% in MPA patients (Log-Rank: p < 0.001; Fig. 1A). The overall survival rate was 87.5% and 77.6% in patients’ first and second periods, respectively (Log-Rank: 0.5). Although there was a trend of higher mortality in the second period compared to the first period, it did not reach statistical significance (p = 0.074) (Table 2). In survival analysis, higher mortality rates were also found in patients who had a severe infection (Log-Rank: p = 0.004; Fig. 1B), active disease at the 6th month (Log-Rank: p < 0.001; Fig. 1C), and had malignancy (Log-Rank: p = 0.035; Fig. 1D).

## Discussion

AAV is still one of the most devastating and potentially lethal forms of autoimmune inflammatory disease [12]. Our study provides a comprehensive analysis of long-term real-life data regarding permanent organ damage and mortality in patients with AAV. Compared to the previously published cohorts, the characteristics of our study permit the capture of relatively rare complications such as malignancy and cardiovascular events [13–15]. Additionally, evaluating organ damage and mortality findings of AAV in different time periods (pre- and post-RTX era) in a large patient population is a novel approach that has not been reported from Türkiye. In our AAV cohort, we observed a favorable survival rate despite significant damage progression. The overall survival rate for GPA was 84.5% in a median of 67.5-month follow-up and was higher from a previous study from our clinic (77% in a median 37-month follow-up) [16]. The five-year survival rate of our cohort was slightly higher compared with the EUVAS study (78.2%) and also comparable with the previously published cohort studies [17, 18] In our cohort, the leading cause of death was severe infection in patients with AAV, as in the previous studies [18–20]. The relatively higher mortality rate in the post-RTX era was remarkable, possibly due to the poor outcomes in patients with COVID-19 who received RTX and developed hypogammaglobulinemia, which was shown in the previous studies [21, 22]. Alamo et al.’s study also showed similar findings regarding mortality rates before and after 2000, consistent with our results [18]. The authors reported the leading causes of mortality as infections (26%), cardiovascular disease (14%), and malignancies (13%), followed by active vasculitis [18]. Although cancer-related mortality was higher in our study, cardiovascular disease-related death was lower compared to the Alamo et al. study. This difference might be due to the lower number of patients in our study.

The development of mortality is associated with cumulative damage in patients with AAV [23]. Although treatment modalities have improved over time, most patients with AAV still develop long-term damage. In a longitudinal analysis of 158 patients with GPA, permanent damage was observed in 86% of patients during the follow-up [24]. In a prospective randomized control trial, the baseline mean score of VDI was 1.3, increasing to 2.5 at the 18-month follow-up [25]. In our cohort, most of the patients (89.7%) had at least one item of VDI, and the average VDI score was consistent with previously published results [26]. A study by Ono et al. revealed that lower VDI scores were recorded in patients who were presented in recent years compared to those followed up earlier, which was consistent with our results [27]. It may be speculated that average VDI scores decreased over time, probably due to the introduction of RTX treatment in AAV by 2011, early recognition and treatment, and relatively reduced CS or CYC doses. The former study also reported the use of lower CS doses in recent years and an association between lower cumulative CS doses and VDI scores. However, our study did not support these findings. This issue may also be due to problems in calculating cumulative CS doses due to the retrospective design of the study. Better outcomes with reduced CS doses compared to standard dose regimens were also observed in the previous studies [19, 28]. Cumulative CYC doses were lower after 2011 in our study due to the higher utility of RTX, as expected.

The average VDI scores were comparable between the two serological subgroups; however, a higher VDI score was observed in patients who could not achieve remission, had relapsed, or died in our study. In our previous study with 50 patients in GPA, the VDI scores were lower in survivors, similar to our present study [16]. In the former study, a correlation between the VDI and BVAS scores was also compatible with the current study. The association between damage and relapse, as well as mortality, was also established in other studies [10, 29].

In our study, the overall development of ESRD was 13.9%, which was lower compared to a recent study (20.6%) [18]. The cumulative incidence of ESRD at 5 and 10 years was 17% and 22%, respectively, in another study [30]. These findings may be due to the longer follow-up duration and higher MPA positivity in the previous studies compared with ours [14]. Peripheral neuropathy as a sequel was 18.1% in our study, similar to the EUVAS group study (15%) [31]. In AAV, the development of malignancy was higher compared to the general population, and cancer was one of the leading causes of death in patients, especially during maintenance treatment [18]. A meta-analysis of Mahr et al. suggests that the standardized incidence ratio of cancer in AAV is 1.6–2.0 compared to the general population with a potentially higher risk in GPA than in MPA [32]. The authors also reported the most frequent malignancies as urinary tract, leukemia, and non-melanoma skin cancer [32]. In our study, the prevalence of malignancy was 8.3% of patients, and one-third were due to genitourinary tract, consistent with the former study. However, the frequency of cancer was similar between patients with GPA and MPA, as well as c-ANCA/PR3 ( +) and p-ANCA/MPO ( +) patients in our study. In addition to the premalignant potential of several immunosuppressives, such as CYC and AZA, chronic immune system stimulation due to AAV may contribute to the increased malignancy risk [33, 34].

Recent studies indicated an increased frequency of CVE in patients with AAV [35, 36]. In our study, the frequency of CVE tended to be higher in patients who had relapsed and could not achieve remission during the induction phase. The contribution of high disease activity to CVE was also shown in a study by Bai et al., which reported that BVAS scores at admission were independently associated with the development of CVE [37]. Berti et al. reported that patients with AAV had a 3 times higher risk for CVE compared to the general population, 6 times higher risk for DVT, and 8.5 times higher risk for CVA [36]. Increased development of CVE might be a manifestation of vasculitis itself, associated with treatments such as CS, or due to accelerated atherosclerosis in patients with AAV. There are few studies establishing an increased frequency of VTE in AAV [36, 38]. Kronbichler et al. found a relationship between high disease activity and VTE [39]. Merkel et al. reported that approximately 10% of GPA patients developed VTE with a median of 2.1 months from enrollment, which was similar (8.8%) to our results [40]. The frequent occurrence of thrombosis in the early phase of treatment in AAV patients may be due to a higher inflammatory state and intense CS treatment during the induction phase.

In our study, the frequency of AVN in patients with AAV was 15.4% and was associated with higher VDI scores. However, only limited data exist regarding the development of AVN in patients with AAV [41, 42]. The high frequency of AVN and its association with damage in patients with AAV may be explained by higher exposure to CS and/or disruption of microcirculation in bone due to underlying vasculitis. Further studies are needed to clarify this issue.

This study has some limitations. The retrospective design of our study, which may have led to missing information for some patients, was the main limitation. The absence of a control group was also a limitation. The retrospective design of the study may have caused a bias in terms of drug choice and outcomes; however, we believe that follow-up of the patients by the same clinicians during the study period would ensure a homogenous treatment approach and data collection.

## Conclusion

Although median VDI scores decreased over the last decade in our AAV cohort, mortality rates remained similar. Mortality was associated with advanced age, diagnosis of MPA (compared to GPA), presence of malignancy, severe infection, higher VDI scores, and active/persistent disease at the 6th month. Patients should be screened for cardiovascular disease and malignancy risk factors, particularly in the genitourinary tract, in patients with AAV. Novel treatment options such as avacopan could help to improve prognosis in patients with AAV.

## Supplementary Information

Below is the link to the electronic supplementary material.

Supplementary file1 (DOCX 24 KB)

## Data Availability

Datasets of the study are available from the corresponding author upon reasonable request.
