# Supplementary material for: Damage accrual and predictors of mortality in ANCA-associated vasculitis: a retrospective observational study
Source: Rheumatol Int. 2025 May 7;45(5):137. doi: 10.1007/s00296-025-05883-7 (PMC12058840; doi:10.1007/s00296-025-05883-7)
Supplement: Supplementary file 1 — Supplementary file1 (DOCX 24 KB) [file 296_2025_5883_MOESM1_ESM.docx]

**Supplementary tables**

**Supplementary Table 1:** Comparison of damage findings of patients between c-ANCA/PR3^+^ and p-ANCA/MPO^+^ according to vasculitis damage index

| VDI Items, n (%) | Total | c-ANCA/PR3^+^ (n=131) | p-ANCA/MPO^+^ (n=88) | p-value (OR) |
| --- | --- | --- | --- | --- |
| Steroid myopathy | 29/232 (12.5) | 15/122 (12.3) | 12/82 (14.6) | 0.6 |
| Osteoporosis | 41/234 (17.5) | 15/122 (12.3) | 19/83 (22.9) | ***0.045 (4)*** |
| Chronic nasal crusting | 16/232 (6.9) | 13/120 (10.8) | 1/84 (1.2) | 0.09 ***^Ɨ^*** |
| Chronic sinusitis | 18/233 (7.7) | 10/121 (8.3) | 3/84 (3.6) | 0.25 ***^Ɨ^*** |
| Avascular necrosis | 35/234 (15) | 23/122 (18.9) | 12/84 (14.3) | 0.4 |
| Cataract | 33/231 (14.3) | 26/121 (21.5) | 5/83 (6) | ***0.003 (9.1) ^Ɨ^*** |
| Blindness (total or partial) | 8/233 (3.4) | 5/121 (4.1) | 1/84 (1.2) | 0.4 ***^Ɨ^*** |
| Hearing loss | 25/232 (10.8) | 20/120 (16.7) | 2/84 (2.4) | ***0.001 (10.5) ^Ɨ^*** |
| Nasal septum perforation | 32/246 (13) | 21/130 (16.2) | 2/85 (2.4) | ***0.001 (10.2) ^Ɨ^*** |
| Trachea/larynx stenosis | 6/246 (2.4) | 4/130 (3.1) | 2/85 (2.4) | 0.8 ***^Ɨ^*** |
| Asthma/chronic dyspnea | 24/236 (10.2) | 8/122 (6.6) | 12/85 (14.1) | 0.07 ***^Ɨ^*** |
| Cardiovascular disease | 38/237 (16) | 25/122 (20.5) | 9/85 (10.6) | 0.06 |
| Myocardial infarction | 14/236 (5.9) | 9/122 (7.4) | 3/84 (3.6) | 0.25 ***^Ɨ^*** |
| Angioplasty | 13/235 (5.5) | 7/122 (5.7) | 4/84 (4.8) | 0.8 ***^Ɨ^*** |
| Valvular heart disease | 5/236 (2.1) | 4/122 (3.3) | 1/84 (1.2) | 0.3 ***^Ɨ^*** |
| Coronary heart disease | 23/236 (9.7) | 15/123 (12.2) | 5/84 (6) | 0.14 ***^Ɨ^*** |
| Cardiomyopathy | 8/237 (3.4) | 4/122 (3.3) | 4/85 (4.7) | 0.6 ***^Ɨ^*** |
| Proteinuria>0.5 g/day | 47/235 (20) | 21/122 (17.2) | 21/84 (25) | 0.17 |
| eGFR<50 ml/min at last visit | 83/238 (34.9) | 42/122 (34.4) | 30/84 (35.7) | 0.85 |
| End-stage renal disease | 33/237 (13.9) | 14/122 (11.5) | 15/85 (17.6) | 0.2 |
| Cerebrovascular accident | 15/237 (6.3) | 8/122 (6.6) | 6/85 (7.1) | 0.9 ***^Ɨ^*** |
| Hypertension | 91/237 (38.4) | 43/122 (35.2) | 37/85 (43.5) | 0.23 |
| Diabetes mellitus | 30/239 (12.6) | 17/122 (13.9) | 10/85 (11.8) | 0.65 |
| Peripheral neuropathy | 43/238 (18.1) | 19/122 (15.6) | 19/85 (22.4) | 0.2 |
| Malignancy | 20/240 (8.3) | 11/123 (8.9) | 3/85 (3.5) | 0.12 ***^Ɨ^*** |
| Venous thrombosis | 21/239 (8.8) | 11/124 (8.9) | 6/85 (7.1) | 0.6 ***^Ɨ^*** |

VDI: Vasculitis damage index, OR: Odds ratio, eGFR: Estimated glomerular filtration rate

* Mann Whitney U test

^Ɨ^Fisher’s exact test

**Supplementary Table 2:** Univariate and multivariate analysis of factors associated with malignancy in patients with ANCA-associated vasculitis

|  | Univariate analysis | | | Multivariate analysis | |
| --- | --- | --- | --- | --- | --- |
| Variables | **Malignancy + (n=20)** | **Malignancy – (n=220)** | **p-value** | **CI % 95 (OR)** | **p-value** |
| Age (years), mean± SD | 60.1±13.4 | 55.3±14.2 | 0.15 |  | NS |
| Gender, female, n (%) | 10 (50) | 121 (55) | 0.7 |  |  |
| Diagnosis, n (%) |  |  |  |  |  |
| GPA (n=175) | 15 (8.6) | 160 (91.4) | 0.8* |  |  |
| MPA (n=64) | 4 (6.3) | 60 (93.8) |  |  |  |
| ANCA status, n (%) |  |  |  |  |  |
| c-ANCA/PR3 (n=123) | 11 (8.9) | 112 (91.1) | 0.16* |  | NS |
| p-ANCA/MPO (n=85) | 3 (3.5) | 82 (96.5) |  |  |  |
| Lung involvement, n (%) | 17/178 (9.6) | 3/60 (5) | 0.4* |  |  |
| Kidney involvement, n (%) | 17/181 (9.4) | 3/58 (5.2) | 0.4* |  |  |
| Cardiovascular disease, n (%) | 8/38 (21) | 10/199 (5) | ***0.003 (11.7)**** | ***2.2-83 (13.4)*** | ***0.005*** |
| Cerebrovascular accident, n (%) | 2/15 (13.3) | 16/222 (7.2) | 0.3* |  |  |
| Avascular necrosis, n (%) | 4/35 (11.4) | 14/199 (7) | 0.3* |  |  |
| Venous thrombosis, n (%) | 3/21 (14.3) | 15/217 (6.9) | 0.2 |  |  |
| BVAS score at admission, mean± SD, range | 21.3±5.5 | 16.6±6.9 | ***0.049*** |  | NS |
| Smoking history (ever), n (%) | 6/53 (11.3) | 8/156 (5.1) | 0.2* |  | NS |
| Baseline creatinine levels, median (IQR) | 2.2 (3.5) | 1.5 (2.4) | 0.18 |  | NS |
| Cumulative steroid dose, median (IQR) | 8.3 (18.2) | 7.5 (8) | 0.4 |  |  |
| Cumulative CYC dose, median (IQR) | 5 (7.4) | 4.5 (7.8) | 0.96 |  |  |
| VDI score, median (IQR) | 3.5 (4) | 2 (2) | ***0.02*** |  | NS |
| Relapse, n (%) | 5/80 (6.3) | 11/150 (7.3) | 0.8 |  |  |
| Remission at the 6^th^ month, n (%) | 8/104 (7.7) | 5/56 (8.9) | 0.8 |  |  |

SD: Standard deviation, IQR: Interquartile range, CI: Confidence interval, GPA: Granulomatous with polyangiitis, MPA: Microscopic polyangiitis, ANCA: Anti-neutrophil cytoplasmic antibody, CYC: Cyclophosphamide, BVAS: Birmingham vasculitis activity score, VDI: Vasculitis damage index, OR: Odds ratio, NS: Not significant

* Mann Whitney U test ^Ɨ^Fisher’s exact test
